# Prevalence and risk factors for chronic pain following cesarean section: a prospective study

**DOI:** 10.1186/s12871-016-0270-6

**Published:** 2016-10-18

**Authors:** Juying Jin, Lihua Peng, Qibin Chen, Dong Zhang, Li Ren, Peipei Qin, Su Min

**Affiliations:** Department of Anesthesiology, the First Affiliated Hospital, Chongqing Medical University, 1 Youyi Road, Chongqing, 400016 China

**Keywords:** Chronic post-surgical pain, Cesarean section, Risk factors, Postoperative pain

## Abstract

**Background:**

Chronic post-surgical pain (CPSP) remains a major clinical problem which may be associated with impaired activities of daily life and decreased health-related quality of life. Although cesarean section is one of the most commonly performed operations, chronic pain after cesarean delivery has not been well-studied. The purpose of this prospective study was to assess the incidence and risk factors of chronic pain at 3, 6 and 12 months after cesarean delivery.

**Methods:**

We prospectively investigated preoperative demographic and psychological factors, intraoperative clinical factors, and acute postoperative pain in a cohort of 527 women undergoing cesarean section. The women were interviewed and completed pain questionnaires after 3, 6 and 12 months. Questions were about pain intensity, frequency, and location, as well as medical treatment and impact on daily living.

**Results:**

The incidence of CPSP at 3, 6 and 12 months after cesarean section was 18.3 %, 11.3 % and 6.8 %, respectively. Most of the women with CPSP experienced mild pain at rest. The incidence of moderate and severe pain on movement was high at 3 month, and then has a significant decrease at 6 and 12 months. CPSP had a negative influence on the activities of daily living. Independent predictors of CPSP at 3 months included higher average pain intensity on movement within 24 h postoperatively, preoperative depression, and longer duration of surgery. At 6 months, more severe pain during movement within 24 h of surgery and preoperative depression were predictive of pain persistence. And 12 months after surgery, only higher average pain score on movement within 24 h following cesarean section was found to be significant associated with CPSP. The three models all showed moderate discrimination and good calibration for the prediction of CPSP at 3, 6 and 12 months postoperatively.

**Conclusions:**

CPSP was not rare in women undergoing cesarean section. Patients with more intense of acute postoperative pain on movement, preoperative depression, and longer surgical time had greater risk for CPSP following surgery.

**Electronic supplementary material:**

The online version of this article (doi:10.1186/s12871-016-0270-6) contains supplementary material, which is available to authorized users.

## Background

Chronic post-surgical pain (CPSP) has been defined by the International Association for the Study of Pain (IASP) as persistent continuously or intermittently for more than 3 months after surgery [1]. CPSP remains a major clinical problem which may be associated with impaired activities of daily life and decreased health-related quality of life. The incidence of CPSP for various common surgical procedures ranges between 10 and 50 % [2–4]. CPSP is not limited to major surgery or major trauma, as even minor operations such as inguinal hernia repair can have significant consequences with regard to development of chronic pain [5, 6].

The cesarean section rate continues to increase in many parts of the world for both medical indications and maternal choice [7–9]. In many Chinese hospitals, the cesarean section rate was more than 40 %, while in some cases, it was up to 80 % [8, 10], which was much higher than the acceptable cesarean rate (5–15 %) in WHO’s guidelines [11]. Current evidence indicates a relatively low incidence of chronic pain after cesarean delivery, with rates ranging between 1 and 18 % [12–18]. Given about 10 million cesarean deliveries annually in China alone, even a small occurrence of CPSP carries important public health consequences [19]. Therefore, great attention should be paid to the consequences associated with this procedure.

Although cesarean section is one of the most commonly performed operations, chronic pain after cesarean section has not been well-studied. For example, the prevalence of chronic pain after cesarean section is usually observed in retrospective studies, and varies considerably from one study to another. Moreover, the number of studies that have examined the risk factors of CPSP after cesarean section is limited. Some possible risk factors that have been identified for CPSP after cesarean delivery include severe acute postoperative pain, previous pain, non-private insurance status and general anesthesia [12–18]. Psychological factors like depression and anxiety have been proved to be predictive of CPSP following orthopedic, spinal and breast cancer surgery [20–22]. However, whether psychological factors play a role in the development of CPSP has not been explored in patients delivered by cesarean section.

Therefore, further research is needed to provide a more comprehensive assessment of CPSP after cesarean section. In addition, factors that might predispose to this type of problem need to be evaluated more closely. The purpose of this prospective study was to assess chronic pain 3, 6 and 12 months after cesarean delivery in a cohort of women in China. We also aim to elucidate the relative contribution of clinical and psychological risk factors for the development of CPSP following cesarean section.

## Methods

### Study design and participants

This prospective observational cohort study was conducted at the First Affiliated Hospital of Chongqing Medical University - a teaching hospital in China. Women hospitalized for cesarean section from July 2014 to December 2014 were recruited on the hospital ward one day before or on the day of surgery, except during weekends and holidays when research interviewers were unavailable. Patients who agreed to participate were interviewed in person preoperatively and 24 h postoperatively by a trained investigator. Telephone follow-up interviews were conducted 3, 6 and 12 months following surgery. Intraoperative information was collected from the patient record by a research assistant. Additional file 1 is the set of questionnaires applied in the present study. Study participants had to be Chinese-speaking aged between 18 and 45 years. The exclusion criteria were history of major psychiatric disorder and inability to undertake a personal or telephone interview. The study was approved by the local ethics committee (registration number: 2013–61), and written informed consent was obtained from all patients.

### Preoperative questionnaire

Study-specific questionnaires concerning demographic and medical variables were given to the patients for self-administration after being admitted to the obstetric ward. Questions about age, gestational age, previous vaginal delivery, cesarean delivery, and pelvic surgery, history of diabetes mellitus and hypertension, and preoperative pain (eg, pain prior to pregnancy or pain during pregnancy) were included.

Body mass index (BMI) was calculated from height and weight measured on admission for surgery.

In addition, standardized instruments were used to measure psychological well-being. The validated Chinese version of the Edinburgh Postnatal Depression Scale (EPDS) was used to capture depressed mood, with higher scores indicating poorer mental health [23]. Although the EPDS was originally developed to assess postnatal depression, the questionnaire has demonstrated to reliably assess depressive symptoms during pregnancy as well. A cut-off of 12 or above was found to have good psychometric properties for a diagnosis of depression among pregnant women [24].

The State Trait Anxiety Inventory (STAI) measures state and trait anxiety. The STAI scale consisted of 40 statements describing various emotional states, which have been translated into Chinese, and its reliability and validity were acceptable [25]. Twenty of these statements demanded the study subjects to describe their emotional reactions in terms of anxiety at a particular moment or period of the time (state anxiety). Statements were scored on a 4-point Likert scale of increasing intensity, from “not at all” to “very much so” (with scores of 1–4 respectively). The other 20 items demanded the subject to describe how they generally feel and response to the threatening situations (trait anxiety). These items were also scored on a 4-point intensity scale, from “almost never” to “almost always”. For the both parts, possible cumulative scores for each scale ranged from 20 (not anxious) to 80 (high anxiety). A cut-off point of scores >40 was selected for both S-and T-STAI [26].

### Surgical variables

Surgery was performed by one of six experienced obstetricians in a standardized protocol. In cesarean section, the incision was performed via a Pfannenstiel incision except in women with a previous vertical skin incision and a transverse lower uterine segment incision. Intraoperative variables obtained from the operating room record surgical approach (pfannenstiel, or lower vertical), operation time and the volume of blood loss.

### Anesthesia variables

All patients followed the anesthesia protocol routinely used in our department. For elective cesarean section, an epidural technique is the most common form of anesthesia. With the patient in the lateral position, the epidural block was performed in the midline at the L1-2 or L2-3 interspace using a loss of resistance technique.

General anesthesia was used in patients with failure of epidural block or with contraindications to epidural anesthesia. Propofol with succinylcholine was given for induction of general anesthesia, with maintenance by sevoflurane or infusion of propofol combined with remifentanil. Rocuronium or vecuronium was given if additional muscle relaxation is needed.

After surgery, postoperative pain relief was provided by intravenous tramadol patient-controlled analgesia via a Rythmic Pump (Micrel, USA) (bolus 20 mg, lockout 10 min, no basal infusion, hourly maximum dose 100 mg). The subjects were monitored every 6 h for 24 h for pain control, tramadol consumption, and relevant side effects.

### Assessment at 24 hours after surgery

Patients were visited within 24 h after surgery. Pain intensity was assessed as average pain at rest and on movement during the past 24 h using a numerical rating scale (NRS), with 0 indicating “no pain” and 10 indicating “worst pain imaginable”.

### Definition of chronic pain

The presence of chronic pain at 3, 6 and 12 months was defined as pain persisting beyond the normal healing time of 3 months, with an NRS value of above 0 at rest and/or with movement. The point prevalence was determined by the proportion of patients reporting chronic pain at the time of the survey.

### Assessment after 3, 6 and 12 months

Patients were contacted by telephone by one of the authors at 3, 6, and 12 months following surgery. This telephone interview usually takes no longer than 15 min. CPSP was measured using the short form Brief Pain Inventory (BPI) [27]. The short form BPI is a self-reporting questionnaire and has been used to describe postoperative pain experiences for adults after major surgery [28]. The BPI consists of four questions related to pain severity and seven questions related to pain interference with function. The pain severity items are presented as NRS. The patients were asked to rate their pain at rest and during movement in the previous week of the survey. The pain severity subscale was also categorized into none (0), mild (1–3), moderate (4–6), and severe (7–10). There were also questions about the location, and the frequency of the pain as well as the consumption of analgesics. The seven items of pain interference on function are also presented as NRS, with 0 = does not interfere and 10 = interferes completely. The interference items ask how pain interferes with general activity, mood, walking, work, relations with others, sleep and enjoyment of life.

### Statistical analysis

Descriptive analyses were conducted to assess the demographic, clinical, and psychological characteristics of the sample. The results are expressed as means ± standard deviation (SD) for continuous variables or as number and percentages for categorical variables.

The initial analyses were conducted to compare women with and without chronic pain at 3, 6 and at 12 months postoperatively to identify risk factors associated with chronic pain after cesarean delivery. For continuous variables, the independent samples *t* test or Mann–Whitney *U* test was used, depending on whether data were normally distributed. For categorical variables, comparison of groups was performed with the *χ* ^2^ test.

Univariate logistic regression analysis was performed to test the influence of possible risk factors on chronic pain at 3, 6 or 12 months after surgery, and candidate covariates were chosen based on statistical significance or possible clinical importance. Only covariates with *P-*values less than 0.25 in the univariate analysis were entered in the multivariate model. In multiple regression, covariates were selected by stepwise backwards likelihood ratio variable selection with P to enter 0.05 and P to remove 0.10. The discriminatory power of the multivariate model was evaluated by using the area under the receiver operating characteristic curve (ROC AUC) and its 95 % confidence interval (CI). An AUC of 0.5 indicates that the model has a predictive discrimination no better than chance, whereas an AUC of 1.0 indicates a perfectly discriminating model. Generally, an AUC of 0.5–0.7 is interpreted as a model with low discriminatory power, 0.7–0.9 moderate and > 0.9 as a model with a high discriminatory power. The calibration of the multivariate model was evaluated using the Hosmer-Lemeshow goodness-of-fit statistic, where a high *P*-value indicates good calibration.

Statistical calculations were performed using SPSS for Windows version 17.0. Two-sided *P*-values of 0.05 were considered statistically significant.

## Results

From July 2014 to December 2014, 597 patients were invited to participate in the study and 32 were declined. Of the 565 patients who were included, 38 were excluded for the following reasons: operation cancelled (*n* = 24), inability to complete the questionnaire because of postoperative complications or intensive care unit admission (*n* = 2), and refusal of the whole inhospital interview (*n* = 12). Hence, 527 patients (94.6 %) who completed the first postoperative questionnaire at 24 h after surgery were included in the analysis. Patient baseline characteristics together with data about surgery and anesthesia are summarized in Table 1.

**Table 1:** Baseline demographic and clinical characteristics (*n* = 527)

Age (year, mean ± SD)	29 ± 4
Body mass index (mean ± SD)	28.3 ± 1.6
Gestational age (week, mean ± SD)	39.0 ± 1.0
Pain prior to pregnancy, n (%)	22 (4.2)
Pain during pregnancy, n (%)	36 (6.8)
Pain with menstruation, n (%)	123 (23.3)
Previous vaginal delivery, n (%)	36 (6.8)
Previous cesarean delivery, n (%)	46 (8.7)
Previous pelvic surgery, n (%)	82 (15.6)
History of diabetes, n (%)	110 (20.9)
History of hypertension, n (%)	36 (6.8)
Number of fetuses (1/2, n (%))	507 (96.2)/20 (3.8)
EPDS score (mean ± SD)	7.8 ± 2.3
sSTAI score (mean ± SD)	38.5 ± 7.5
tSTAI score (mean ± SD)	39.3 ± 7.8
General anesthesia/epidural anesthesia, n (%)	44 (8.3)/483 (91.7)
Elective/emergent, n (%)	294 (55.8)/233 (44.2)
Duration of surgery (min, mean ± SD)	43 ± 7
Vertical/Pfannenstiel incision, n (%)	17 (3.2)/510 (96.8)
Blood loss (mL, mean ± SD)	197 ± 99
Average pain intensity at rest within 24 h postoperatively	1.8 ± 0.5
Average pain intensity on movement within 24 h postoperatively	3.3 ± 0.7

*SD* standard deviation, *EPDS* Edinburgh postnatal depression scale, *sSTAI* state-State Trait Anxiety Inventory, tSTAI trait-State Trait Anxiety Inventory

The response rates for the questionnaires in these 527 patients after 3, 6, and 12 months were 502 (95.3 %), 487 (92.4 %), and 472 (89.6 %), respectively. Using the primary study definition, 92 of 502 patients (18.3 %) reported characteristic of CPSP 3 months after surgery. Rates are based on any symptom of pain, ache, or discomfort in the operated area that was experienced in the previous week of the survey. By 6 months postoperatively, the observed rate of pain fell to 55 of 487 (11.3 %). At 12-month follow-up only 32 of 472 patients (6.8 %) still complained of persisting postoperative pain. Of the subjects with persistent pain, the timing and severity are shown in Table 2.

**Table 2:** Characteristics of pain at 3, 6 and 12 months following surgery

	Pain at 3 months *n* = 92	Pain at 6 months *n* = 55	Pain at 12 months *n* = 32
Pain severity			
Pain at rest in past week			
NRS score (mean ± SD)	2.7 ± 1.1	2.0 ± 1.2	1.4 ± 1.0
No pain	2 (2.2 %)	12 (21.8 %)	9 (28.1)
Mild pain (n, %)	67 (72.8 %)	40 (72.7 %)	23 (71.9 %)
Moderate pain (n, %)	23 (25 %)	3 (5.5 %)	0
Severe pain (n, %)	0	0	0
Pain during movement in past week			
NRS score (mean ± SD)	4.4 ± 1.1	3.5 ± 1.3	2.4 ± 0.8
Mild pain (n, %)	19 (20.7 %)	36 (65.5 %)	31 (96.9 %)
Moderate pain (n, %)	68 (73.9 %)	17 (30.9 %)	1 (3.1 %)
Severe pain (n, %)	5 (5.4 %)	2 (3.6 %)	0
Analgesic required (n, %)	25 (27.2 %)	10 (18.2 %)	7 (21.9 %)
Pain frequency (n, %)			
Constantly	16 (17.4 %)	7 (12.7 %)	1 (3.1 %)
Daily	26 (28.3 %)	9 (16.4 %)	1 (3.1 %)
Several times/week	27 (29.3 %)	16 (29.1 %)	10 (31.3 %)
Once a week	17 (18.5 %)	11 (20 %)	13 (40.6 %)
Less than once a week	6 (6.5 %)	12 (21.8 %)	7 (21.9 %)
Main location of pain (n, %)			
Area of incision	41 (44.6 %)	18 (32.7 %)	8 (25 %)
Pelvis	23 (25 %)	20 (36.4 %)	12 (37.5 %)
Low back	12 (13.0 %)	6 (10.9 %)	6 (18.8 %)
Buttocks	9 (9.8 %)	7 (12.7 %)	3 (9.4 %)
Legs	7 (7.6 %)	4 (7.3 %)	3 (9.4 %)

*NRS* numerical rating scale, *SD* standard deviation

Most women with chronic pain reported mild pain at rest during the first year after giving birth. The incidence of moderate to severe chronic pain at rest was 25 % (23 of 92), 5.5 % (3 of 55) and 0 among mothers reporting chronic pain at 3, 9 and 12 months, respectively. At 3, 6 and 12 months postoperatively, approximately 80 %, 35 % and 3 % of those with persistent pain reported moderate to severe pain on movement. Among the patients with CPSP at 3 month after surgery, only a small proportion of them reported having taken analgesics (27.2 %). And 6 months and 12 months after surgery, 18.2 % and 21.9 % of the patients with CPSP reported having used analgesic medication during the week preceding the survey, respectively.

At 3 months, pain was present daily or several times per week in majority of the patients with CPSP. At 6 months, pain was present several times per week or once a week in about half of the patients with CPSP, and by 12 months postoperatively pain occurred once a week or less than once a week in nearly two thirds patients who reported CPSP. The two most common sites of CPSP were the area of incision and the pelvis. Further locations affected were low back, buttocks and legs (Table 2).

At 3 months, general activity was the domain most affected by pain, with the majority (84.8 %) of individuals with pain reporting that this aspect of their life was affected (Table 3). Normal work appeared to be the activity least affected by pain, with 66.3 % of patients stating that their pain had an impact on normal work. At 6 months and 12 months, the interference of pain was important on mood and enjoyment of life. The impact of pain on normal work, relations with other people or on sleep was less important.

**Table 3:** The activities of women’s daily living affected by chronic pain, and the extent of perceived interference

Activity	3 months after surgery (*n* = 92)		6 months after surgery (*n* = 55)		12 months after surgery (*n* = 32)	
	Women who stated that pain had some effect on this activity (*n*, %)	NRS score (mean ± SD)	Women who stated that pain had some effect on this activity (*n*, %)	NRS score (mean ± SD)	Women who stated that pain had some effect on this activity (*n*, %)	NRS score (mean ± SD)
General activity	78 (84.8 %)	3.9 ± 1.9	37 (67.3 %)	2.0 ± 1.7	22 (68.8 %)	2.2 ± 1.7
Mood	72 (78.3 %)	3.8 ± 2.3	42 (76.4 %)	2.5 ± 1.7	23 (71.9 %)	2.0 ± 1.6
Walking ability	68 (73.9 %)	2.9 ± 1.9	37 (67.3 %)	1.6 ± 1.5	21 (65.6 %)	1.8 ± 1.8
Normal work	61 (66.3 %)	2.7 ± 2.1	36 (65.5 %)	1.2 ± 1.2	17 (53.1 %)	1.9 ± 1.1
Relations with other people	68 (73.9 %)	2.8 ± 1.9	34 (61.8 %)	1.5 ± 1.5	18 (56.3 %)	1.3 ± 1.5
Sleep	67 (72.8 %)	3.0 ± 2.1	34 (61.8 %)	1.8 ± 1.9	18 (56.3 %)	1.7 ± 1.8
Enjoyment of life	71 (77.2 %)	3.8 ± 2.4	40 (72.7 %)	2.3 ± 1.8	24 (75.0 %)	2.4 ± 1.9

Table 4 presents demographic, clinical, and preoperative emotional functioning variables for women with and without chronic pain at 3, 6 and 12 months. Factors with a statistically significant association with chronic pain at 3 months included previous cesarean delivery, preoperative depression (EPDS score ≥ 12), longer duration of surgery, higher pain scores both at rest and during movement within 24 h postoperatively. At 6 months following cesarean delivery, in addition to previous risks factors for CPSP at 3 months postoperatively, type of anesthesia was also found to significantly differ between patients who did and did not develop chronic pain. Both risks factors for CPSP at 6 months and intraoperative blood loss volume were significantly associated with chronic pain at 12 months.

**Table 4:** Demographic, clinical, and psychological factors associated with chronic pain at 3, 6 and 12 months after Cesarean section

	3 months			6 months			12 months		
	Chronic pain (*n* = 92)	No chronic pain (*n* = 410)	*P* value	Chronic pain (*n* = 55)	No chronic pain (*n* = 432)	*P* value	Chronic pain (*n* = 32)	No chronic pain (*n* = 440)	*P* value
Age (year, mean ± SD)	30 ± 4	29 ± 4	0.354	29 ± 4	29 ± 4	0.780	30 ± 4	29 ± 4	0.346
Body mass index (mean ± SD)	28.4 ± 1.7	28.3 ± 1.6	0.400	28.3 ± 1.8	28.3 ± 1.6	0.803	28.5 ± 1.7	28.3 ± 1.6	0.432
Gestational age (week, mean ± SD)	38.9 ± 1.3	39.0 ± 1.0	0.469	38.9 ± 1.4	39.0 ± 1.0	0.458	38.9 ± 1.5	39.0 ± 1.0	0.406
Pain prior to pregnancy, *n* (%)			0.986			0.334			0.230
Yes	4 (4.3 %)	18 (4.4 %)		1 (1.8 %)	20 (4.6 %)		0	19 (4.3 %)	
No	88 (95.7 %)	392 (95.6 %)		54 (98.2 %)	412 (95.4 %)		32 (100 %)	421 (95.7 %)	
Pain during pregnancy, *n* (%)			0.241			0.062			0.205
Yes	9 (9.8 %)	26 (6.3 %)		7 (12.7 %)	26 (6.0 %)		4 (12.5 %)	29 (6.6 %)	
No	83 (90.2 %)	384 (93.7 %)		48 (87.3 %)	406 (94.0 %)		28 (87.5 %)	411 (93.4 %)	
Pain with menstruation, *n* (%)			0.634			0.966			0.117
Yes	23 (25.0 %)	93 (22.7 %)		13 (23.6 %)	101 (23.4 %)		11 (34.4 %)	98 (22.3 %)	
No	69 (75.0 %)	317 (77.3 %)		42 (76.4 %)	331 (76.6 %)		21 (65.6 %)	342 (77.7 %)	
Previous vaginal delivery, *n* (%)			0.916			0.769			0.980
Yes	6 (6.5 %)	28 (6.8 %)		3 (5.5 %)	28 (6.5 %)		2 (6.3 %)	28 (6.4 %)	
No	86 (93.5 %)	382 (93.2 %)		52 (94.5 %)	404 (93.5 %)		30 (93.8 %)	412 (93.6 %)	
Previous cesarean delivery, *n* (%)			0.012			0.037			0.009
Yes	14 (15.2 %)	29 (7.1 %)		9 (16.4 %)	34 (7.9 %)		7 (21.9 %)	36 (8.2 %)	
No	78 (84.8 %)	381 (92.9 %)		46 (83.6 %)	398 (92.1 %)		25 (78.1 %)	404 (91.8 %)	
Previous pelvic surgery, *n* (%)			0.572			0.648			0.229
Yes	12 (13.0 %)	63 (15.4 %)		7 (12.7 %)	65 (15.0 %)		7 (21.9 %)	62 (14.1 %)	
No	80 (87.0 %)	347 (84.6 %)		48 (87.3 %)	367 (85.0 %)		25 (78.1 %)	378 (85.9 %)	
History of diabetes, *n* (%)			0.656			0.602			0.337
Yes	21 (22.8 %)	85 (20.7 %)		13 (23.6 %)	89 (20.6 %)		9 (28.1 %)	92 (20.9 %)	
No	71 (77.2 %)	325 (79.3 %)		42 (76.4 %)	343 (79.4 %)		23 (71.9 %)	348 (79.1 %)	
History of hypertension, *n* (%)			0.857			0.602			0.230
Yes	7 (7.6 %)	29 (7.1 %)		5 (9.1 %)	31 (7.2 %)		4 (12.5 %)	30 (6.8 %)	
No	85 (92.4 %)	381 (92.9 %)		50 (90.9 %)	401 (92.8 %)		28 (87.5 %)	410 (93.2 %)	
Number of fetuses, *n* (%)			0.370			0.914			0.788
1	90 (97.8 %)	393 (95.9 %)		53 (96.4 %)	415 (96.1 %)		31 (96.9 %)	422 (95.9 %)	
2	2 (2.2 %)	17 (4.1 %)		2 (3.6 %)	17 (3.9 %)		1 (3.1 %)	18 (4.1 %)	
EPDS score (mean ± SD)			<0.001			<0.001			0.007
< 12	74 (80.4 %)	393 (95.9 %)		42 (76.4 %)	411 (95.1 %)		26 (81.3 %)	413 (93.9 %)	
≥ 12	18 (19.6 %)	17 (4.1 %)		13 (23.6 %)	21 (4.9 %)		6 (18.8 %)	27 (6.1 %)	
sSTAI score (mean ± SD)			0.321			0.500			0.782
≤ 40	60 (65.2 %)	289 (70.5 %)		36 (65.5 %)	302 (69.9 %)		23 (71.9 %)	306 (69.5 %)	
> 40	32 (34.8 %)	121 (29.5 %)		19 (34.5 %)	130 (30.1 %)		9 (28.1 %)	134 (30.5 %)	
tSTAI score (mean ± SD)			0.306			0.245			0.944
≤ 40	51 (55.4 %)	251 (61.2 %)		29 (60.0 %)	263 (60.9 %)		19 (59.4 %)	264 (60.0 %)	
> 40	41 (44.6 %)	159 (38.8 %)		26 (47.3 %)	169 (39.1 %)		13 (40.6 %)	176 (40.0 %)	
Type of anesthesia, *n* (%)			0.089			0.007			0.031
General anesthesia	12 (13.0 %)	31 (7.6 %)		10 (18.2 %)	32 (7.4 %)		6 (18.8 %)	34 (7.7 %)	
Epidural anesthesia	80 (8.7 %)	379 (92.4 %)		45 (81.8 %)	400 (92.6 %)		26 (81.3 %)	406 (92.3 %)	
Type of surgery, *n* (%)			0.505			0.145			0.274
Elective	49 (53.3 %)	234 (57.1 %)		36 (65.5 %)	238 (55.1 %)		21 (65.6 %)	245 (55.7 %)	
Emergent	43 (46.7 %)	176 (42.9 %)		19 (34.5 %)	194 (44.9 %)		11 (34.4 %)	195 (44.3 %)	
Duration of surgery (min, mean ± SD)	46.8 ± 8.2	42.3 ± 6.8	<0.001	47.0 ± 7.0	42.6 ± 7.2	<0.001	48.4 ± 6.8	42.7 ± 7.1	<0.001
Type of incision, *n* (%)			0.965			0.565			0.986
Vertical	3 (3.3 %)	13 (3.2 %)		1 (1.8 %)	14 (3.2 %)		1 (3.1 %)	14 (3.2 %)	
Pfannenstiel	89 (96.7 %)	397 (96.8 %)		54 (98.2 %)	418 (96.8 %)		31 (96.9 %)	426 (96.8 %)	
Blood loss (mL, mean ± SD)	212 ± 126	193 ± 94	0.103	221 ± 130	194 ± 97	0.058	255 ± 152	193 ± 96	0.001
Average pain intensity at rest within 24 h postoperatively (mean ± SD)	2.0 ± 0.6	1.7 ± 0.5	<0.001	2.1 ± 0.6	1.7 ± 0.5	<0.001	2.1 ± 0.6	1.7 ± 0.5	<0.001
Average pain intensity on movement within 24 h postoperatively (mean ± SD)	3.7 ± 0.9	3.2 ± 0.7	<0.001	3.9 ± 0.8	3.2 ± 0.8	<0.001	3.9 ± 0.8	3.2 ± 0.7	<0.001

As Table 5 shows, 8, 10 and 12 variables were included in the subsequent multiple logistic regression models predicting chronic pain at 3, 6 and 12 months respectively, because *P* < 0.25 for the comparisons between patients who did and did not develop chronic pain. As Table 5 shows, three significant risk factors were identified for CPSP of 3 months after surgery: preoperative depression (OR 4.641, 95 % CI 2.078-10.363, *P* < 0.001), higher average pain intensity on movement within 24 h postoperatively (OR 2.396, 95 % CI 1.622-3.539, *P* < 0.001), and longer duration of surgery (OR 1.065, 95 % CI 1.024-1.107, *P* = 0.001). At 6 months postoperatively, preoperative depression and more severe pain during movement within 24 h of surgery were found to independently associate with occurrence of CPSP. At 12 months, only average pain score on movement within 24 h following cesarean section remained significantly associated with CPSP in the multivariate analysis (OR 2.743, 95 % CI 1.453-5.178, *P* = 0.002).

**Table 5:** Logistic regression model for presence of chronic pain following cesarean section

	3 months (*n* = 502)				6 months (*n* = 487)			12 months (*n* = 472)		
		Odds Ratio	95 % Confidential Interval	*P*	Odds Ratio	95 % Confidential Interval	*P*	Odds Ratio	95 % Confidential Interval	*P*
Pain prior to pregnancy	No							1		0.998
	Yes								0	
Pain during pregnancy	No	1		0.697	1		0.531	1		0.955
	Yes	1.199	0.482-2.982		0.719	0.257-2.016		1.038	0.285-3.781	
Pain with menstruation	No							1		0.094
	Yes							0.473	0.197-1.136	
Previous cesarean delivery	No	1		0.087	1		0.151	1		0.147
	Yes	0.513	0.239-1.102		0.509	0.203-1.280		0.448	0.151-1.328	
Previous pelvic surgery	No							1		0.061
	Yes							0.367	0.128-1.048	
History of hypertension	No							1		0.744
	Yes							0.744	0.194-2.856	
EPDS score	<12	1		<0.001	1		<0.001	1		0.179
	≥12	4.641	2.078-10.363		5.526	2.192-13.931		2.300	0.682-7.760	
tSTAI score	≤40				1		0.530			
	>40				1.227	0.648-2.321				
Type of anesthesia	General	1		0.284	1		0.063	1		0.068
	Epidural	0.645	0.290-1.438		0.431	0.178-1.046		0.346	0.111-1.081	
Type of surgery	Elective				1		0.176			
	Emergent				1.588	0.813-3.102				
Duration of surgery		1.065	1.024-1.107	0.001	1.034	0.985-1.086	0.172	1.054	0.993-1.119	0.083
Blood loss		0.999	0.996-1.002	0.522	1.000	0.997-1.004	0.782	1.002	0.999-1.006	0.169
Average pain intensity at rest within 24 h postoperatively		1.067	0.636-1.789	0.806	1.442	0.744-2.792	0.278	2.108	0.876-5.071	0.096
Average pain intensity on movement within 24 h postoperatively		2.396	1.622-3.539	<0.001	2.509	1.557-4.043	<0.001	2.743	1.453-5.178	0.002

The predictive models for CPSP at 3, 6 and 12 months yielded ROC AUC of 0.708 (95 % CI 0.641 - 0.755), 0.726 (95 % CI 0.646 - 0.805) and 0.744 (95 % CI 0.648- 0.840), respectively. And the three models showed good calibration by Hosmer-Lemeshow goodness-of-fit statistic with *χ* ^2^ = 9.542, *P* = 0.299 (3 months), *χ* ^2^ = 5.395, *P* = 0.715 (6 months) and *χ* ^2^ = 5.862, *P* = 0.663 (12 months), respectively. This indicates that the models had moderate discriminatory power and good calibration.

## Discussion

In our prospective cohort study of 527 women who underwent cesarean delivery, the incidence of CPSP was 18.3 % at 3 months after surgery. And we found 11.3 % of patients still had pain 6 months following surgery, and the incidence of CPSP subsequently reduced to 6.8 % at 12 months. In 2004 Nikolajsen et al. [12] published the first report on the incidence of chronic pain after cesarean delivery in a cohort of Danish women. They reported a CPSP incidence of 18.6 % at 3 months and 12.3 % of the patients experienced persistent pain at the end of a follow-up period ranging from 6 to 18 months. In another Asian retrospective study, the incidence of chronic pain after 3 months was 9.2 % after elective cesarean section under spinal anesthesia [13]. Liu et al. [18] reported that the incidence of persistent pain following cesarean section at 2 months was 15 %, and it subsequently reduced to 4 % at 12 months. Eisenach et al. [17] undertook a prospective longitudinal cohort study to describe the incidence of acute and chronic pain after delivery. They reported a surprisingly low incidence of CPSP after delivery, as low as 1.8 % at 6 months and 0.3 % at 12 months. However, in this study, only those individuals who reported pain at 2 months after delivery were followed up at 6 months, and similarly, only those who reported pain at 6 months were contacted at 12 months. Such a protocol assumes that CPSP is a continuum from acute postoperative pain; this assumption may be far from the reality. Indeed, other surgical procedures suggest that CPSP may develop later after surgery [29]. In the current report, it was not always the same patients who reported pain at 3, 6 and 12 months. Patients with low levels of pain may be pain-free at one measurement and may experience pain at the next measurement, showing the fluctuation of pain. This supports the notion that chronic pain may be a constantly changing outcome, rather than a static trait. Therefore, the study from Eisenach might have missed some patients who developed pain in their late follow-up. Overall, the wide interval of reported prevalence of CPSP may be explained by discrepancies in terms of methodology, chronic pain definition, time after surgery, and finally anesthetic and analgesic management.

As indicated, the prevalence of CPSP is highly dependent on its definition. We defined CPSP as pain that persists beyond 3 months with an NRS value of above 0 after surgery using the IASP’s recommendations [1]. A specific definition for chronic pain after surgery was proposed by Macrae and Davies which specified that pain should develop after surgery, have lasted for at least 2 months and other causes for the pain should be excluded [2]. However, causality is difficult to prove, particularly within large-scale studies where it is not possible to conduct detailed clinical investigation of individuals. A pragmatic decision was therefore taken to use the widely accepted IASP definition as has been used in other studies investigating CPSP [12, 13, 30, 31].

We found most of the women with CPSP experienced mild pain at rest, few women experienced severe or unbearable rest pain. Moderate and severe pain on movement was frequent 3 month following cesarean section, whereas the incidence of moderate to severe pain on movement significantly decreased at 6 and 12 months. The severity of the pain reported by patients in the present study with chronic pain was comparable to most other studies [12, 13, 15, 18]. However, due to the use of different instruments for pain assessment, the comparison between studies should be performed with caution.

The present study found that CPSP had a negative influence on the activities of daily living of patients undergoing cesarean section. Physical and social consequences of chronic pain were predominated in general activity, mood and enjoyment of life. This exemplifies the impact of chronic pain on the mental health of this relatively young population. Moreover, there seems to be a psychic component that explains some repercussions of chronic pain.

One of our objectives was to identify demographic, clinical, and psychological risk factors for chronic pain after cesarean section. The results of our multivariate analyses indicate that patients with higher average pain intensity on movement within 24 h postoperatively have a greater risk of chronic pain at 3, 6 and 12 months after cesarean section. Therefore, our results indicate that attempts to better manage postoperative pain are needed in CPSP prevention.

Substantial evidence has been generated over the last decade suggesting that severe acute pain after delivery and specifically after cesarean delivery may progress to chronic pain [12, 13, 15, 16]. Moreover, acute postoperative pain has been proved to be one of the most consistent and strongest predictors of CPSP after a range of other surgery procedures, including hernia repair [32], thoracotomy [33], limb amputation [34], and coronary artery bypass [35]. A retrospective study undertaken in Singapore found CPSP was significantly favored by higher early postoperative pain after cesarean section under spinal anesthesia [13]. In a cohort study, CPSP was showed to be associated with early postoperative pain intensity [15]. Acute pain indicates actual or potential tissue damage and motivates a response that removes the organism from that threat. Activation of these peripheral nociceptors signals the presence, location, intensity and duration of a noxious stimulus and fades when the stimulus is removed [36, 37].

It has been shown that depression during pregnancy is associated with increased maternal mortality [38]. The present study indicates that preoperative depression strongly predicts chronic pain at 3 and 6 months after surgery. This may imply that patients likely to develop chronic pain after cesarean delivery are a subset of a vulnerable group of patients more likely to report pain. A previous systematic review has been indicated that depression represents a risk factor for the development of chronic pain following various surgical procedures [39]. Hobson et al. [40] found that lower preoperative depression is associated with greater maternal satisfaction and better recovery after elective cesarean section. It makes sense that effective management of preoperative depression would be crucial for prevention of CPSP.

In this study, the duration of the surgery is found to be associated with CPSP at 3 months following cesarean section. Previous studies have not evaluated this variable as a risk factor for CPSP following cesarean section. As operations with longer duration may be associated with more extensive tissue injury during surgery, there is a higher risk of damaging nerves in the area that may increase the risk of subsequent chronic pain [41, 42].

Unlike Nikolajsen et al. [12], we did not find a significant correlation between general anesthesia and persistent pain, although this element may also be underpowered, as only 8.3 % of our patients had undergone general anesthesia. And it is not supported by either our study or that of Eisenach et al. [16] that general anesthesia leads to a higher incidence of chronic pain compared with intrathecal anesthesia.

Previous surgery has been recognized as a risk factor for chronic pain in general after surgery [4]. In the present study, previous cesarean delivery was associated with chronic pain in univariate but not in multiple logistic regression analyses. However, previous cesarean section as a risk factor for chronic pain deserves further attention.

Our study has limitations and strengths. The present study relied on the patients’ selfreport, and physical examinations, quantitative sensory testing, or both, were not undertaken. This may be seen as a limitation of our study. Another limitation of this study was that we did not have the ability to examine the effect of nonresponse because we did not have permission to access information on prospective subjects who declined to participate. However, characteristics of our sample are similar to other reports in the literature. Age, BMI, and previous pelvic surgery status were similar between our sample and the literature. A final limitation of the current study was that our sample was drawn from a single teaching hospital, and all the women in our study were Asian, that is, we do not know if the figures can be generalised to other settings. Therefore, it would be important to evaluate the generalizability of our findings to more heterogeneous samples of patients.

Despite these limitations, methodologic features of our study and its results contribute to knowledge of chronic pain following cesarean section. The strengths of the study are the prospective design, the consecutive inclusion of patients and the use of validated tools for symptom measurements. Unlike retrospective studies relying on patient recall of events occurring months or even one year previously, patients in our study were asked to report pain at the time of their interview, rather than recalling previous pain experiences. The study included 90 % of all patients operated on during the study period and had high response rates at follow-up. The inclusion of patients was restricted to 6 months; consequently, the surgical procedures and post-operative pain management were similar for all patients.

## Conclusions

In summary, we found that the incidence of CPSP in patients undergoing cesarean section was 18.3 % at 3 months, and subsequently reduced to 6.8 % at 12 months. Pain intensity was mild to moderate in most of women with CPSP. Predictors for CPSP included higher average intensity score of acute postoperative pain on movement, preoperative depression and longer surgical duration. Preventive strategies should target these risk factors to reduce adverse sequelae of procedure, supplemented with broader efforts to support the longer-term recovery in parturient.

## Additional file

Additional file 1: The set of questionnaires. (DOCX 131 kb)

## Abbreviations

BPI: Brief pain inventory
CPSP: Chronic post-surgical pain
EPDS: Edinburgh Postnatal Depression Scale
IASP: International Association for the Study of Pain
NRS: Numerical rating scale
SD: Standard deviation
SPSS: Statistical package for the social sciences
sSTAI: state-State Trait Anxiety Inventory
tSTAI: trait-State Trait Anxiety Inventory
