# Supplementary material for: Prevalence and risk factors for chronic pain following cesarean section: a prospective study
Source: BMC Anesthesiol. 2016 Oct 18;16:99. doi: 10.1186/s12871-016-0270-6 (PMC5069795; doi:10.1186/s12871-016-0270-6)
Supplement: Additional file 1: — The set of questionnaires. (DOCX 131 kb) [file 12871_2016_270_MOESM1_ESM.docx]

**Questionnaire translated into English**

**Preoperative Questionnaire**

Date of (MM/DD/YY)

Patient Name

Patient ID

Phone Number for follow-up contact

1. Patient date of birth (MM/DD/YY)

2. Patient height (cm)

3. Patient weight (kg)

4. Gestational age (weeks)

5. Number of fetuses

6. Have you had a Cesarean section previously?

□Yes

□No

7. Have you had a vaginal delivery previously?

□Yes

□No

8. Have you had any other pelvic operations previously?

□Yes

□No

If yes, please specify:

9. Did you have chronic pain (last for more than 3 months) before this pregnancy?

□Yes

□No

10. Have you had chronic pain (last for more than 3 months) during this pregnancy?

□Yes

□No

11. Did you usually have pain with your menstrual bleeding?

□Yes

□No

12. Did you smoke cigarettes (more than 1 cigarette per day for at least 6 months) before this pregnancy?

□Yes

□No

13. Did you drink alcohol (at least 30g of alcohol per week for at least 1 year) before this pregnancy?

□Yes

□No

14. Have you been diagnosed with high blood pressure before or during this pregnancy?

□Yes

□No

15. Have you been diagnosed with diabetes before or during this pregnancy?

□Yes

□No

**Edinburgh Postnatal Depression Scale**

Please check the answer that comes closest to how you have felt IN THE PAST 7 DAYS, not just how you feel today.

Here is an example, already completed.

I have felt happy:

□Yes, all the time

Yes, most of the time

□No, not very often

No, not at all

This would mean: “I have felt happy most of the time” during the past week. Please complete the other questions in the same way.

In the past 7 days:

1. I have been able to laugh and see the funny side of things

□As much as I always could

□Not quite so much now

□Definitely not so much now

□Not at all

2. I have looked forward with enjoyment to things

□As much as I ever did

□Rather less than I used to

□Definitely less than I used to

□Hardly at all

3. I have blamed myself unnecessarily when things went wrong

□Yes, most of the time

□Yes, some of the time

□Not very often

□No, never

4. I have been anxious or worried for no good reason

□No, not at all

□Hardly ever

□Yes, sometimes

□Yes, very often

5. I have felt scared or panicky for no very good reason

□Yes, quite a lot

□Yes, sometimes

□No, not much

□No, not at all

6. Things have been getting on top of me

□Yes, most of the time I haven’t been able to cope at all

□Yes, sometimes I haven’t been coping as well as usual

□No, most of the time I have coped quite well

□No, have been coping as well as ever

7. I have been so unhappy that I have had difficulty sleeping

□Yes, most of the time

□Yes, sometimes

□Not very often

□No, not at all

8. I have felt sad or miserable

□Yes, most of the time

□Yes, quite often

□Not very often

□No, not at all

9. I have been so unhappy that I have been crying

□Yes, most of the time

□Yes, quite often

□Only occasionally

□No, never

10. The thought of harming myself has occurred to me

□Yes, quite often

□Sometimes

□Hardly ever

□Never

**State Trait Anxiety Inventory**

Read each statement and select the appropriate response to indicate how you feel right now, that is, at this very moment. There are no right or wrong answers. Do not spend too much time on any one statement but give the answer which seems to describe your present feelings best.

1 2 3 4

Not at all A little Somewhat Very Much So

1. I feel calm 1 2 3 4

2. I feel secure 1 2 3 4

3. I feel tense 1 2 3 4

4. I feel strained 1 2 3 4

5. I feel at ease 1 2 3 4

6. I feel upset 1 2 3 4

7. I am presently worrying over possible misfortunes 1 2 3 4

8. I feel satisfied 1 2 3 4

9. I feel frightened 1 2 3 4

10. I feel uncomfortable 1 2 3 4

11. I feel self-confident 1 2 3 4

12. I feel nervous 1 2 3 4

13. I feel jittery 1 2 3 4

14. I feel indecisive 1 2 3 4

15. I am relaxed 1 2 3 4

16. I feel content 1 2 3 4

17. I am worried 1 2 3 4

18. I feel confused 1 2 3 4

19. I feel steady 1 2 3 4

20. I feel pleasant 1 2 3 4

1 2 3 4

Almost never Sometimes Often Almost always

21. I feel pleasant 1 2 3 4

22. I feel nervous and restless 1 2 3 4

23. I feel satisfied with myself 1 2 3 4

24. I wish I could be as happy as others seem to be 1 2 3 4

25. I feel like a failure 1 2 3 4

26. I feel rested 1 2 3 4

27. I am calm, cool and collected 1 2 3 4

28. I feel that difficulties are piling up 1 2 3 4

so that I cannot overcome them

29. I worry too much over something 1 2 3 4

that really doesn’t matter

30. I am happy 1 2 3 4

31. I have disturbing thoughts 1 2 3 4

32. I lack self-confidence 1 2 3 4

33. I feel secure 1 2 3 4

34. I make decisions easily 1 2 3 4

35. I feel inadequate 1 2 3 4

36. I am content 1 2 3 4

37. Some unimportant thought runs 1 2 3 4

through my mind and bothers me

38. I take disappointments so keenly 1 2 3 4

that I can’t put them out of my mind

39. I am a steady person 1 2 3 4

40. I get in a state of tension or turmoil 1 2 3 4

over my recent concerns and interests

**Surgical and anesthesia information**

Date of (MM/DD/YY) (MM/DD/YY)

Patient Name

Patient ID

1. Type of anesthesia

□general

□epidural

1. Nature of surgery

□Elective

□Emergent

3. Type of incision

□Pfannenstiel

□vertical

1. Duration of surgery (min)
2. Estimated blood loss (ml)

**Questionnaire for postoperative pain at 24 hours after surgery**

Date of (MM/DD/YY) (MM/DD/YY)

Patient Name

Patient ID

1. Please rate your pain at rest by marking the box beside the number that best describes your pain on the average in the last 24 hours.

□0 □1 □2 □3 □4 □5 □6 □7 □8 □9 □10

(no (worst pain

pain) imaginable)

1. Please rate your pain on movement by marking the box beside the number that best describes your pain on the average in the last 24 hours.

□0 □1 □2 □3 □4 □5 □6 □7 □8 □9 □10

(no (worst pain

pain) imaginable)

**Three-month follow-up questionnaire**

Date of (MM/DD/YY) (MM/DD/YY)

Patient Name

Patient ID

1. Throughout our lives, most of us have had pain from time to time (such as minor headaches, sprains, and toothaches). Have you had pain other than these everyday kinds of pain today?

□Yes

□No

2. On the diagram, shade in the areas where you feel pain. Put an X on the area that hurts the most.


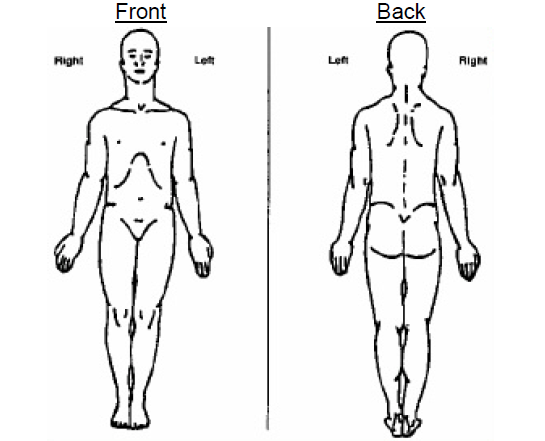


3. Please rate your pain by marking the box beside the number that best describes your pain at rest in the past week.

□0 □1 □2 □3 □4 □5 □6 □7 □8 □9 □10

(no (worst pain

pain) imaginable)

4. Please rate your pain by marking the box beside the number that best describes your pain on movement in the past week.

□0 □1 □2 □3 □4 □5 □6 □7 □8 □9 □10

(no (worst pain

pain) imaginable)

1. What treatments or medications are you receiving for your pain?

6. In the last 24 hours, how much relief have pain treatments or medications provided? Please mark the box below the percentage that most shows how much relief you have received.

□0% □10% □20% □30% □40% □50% □60% □70% □80% □90% □10%

(no (complete

relief) relief)

7. Mark the box beside the number that describes how, during the past 24 hours, pain has interfered with your:

A. General Activity

□0 □1 □2 □3 □4 □5 □6 □7 □8 □9 □10

(Does not (completely

interfere) interferes)

B. Mood

□0 □1 □2 □3 □4 □5 □6 □7 □8 □9 □10

(Does not (completely

interfere) interferes)

C. Walking ability

□0 □1 □2 □3 □4 □5 □6 □7 □8 □9 □10

(Does not (completely

interfere) interferes)

D. Normal Work (includes both work outside the home and housework)

□0 □1 □2 □3 □4 □5 □6 □7 □8 □9 □10

(Does not (completely

interfere) interferes)

E. Relations with other people

□0 □1 □2 □3 □4 □5 □6 □7 □8 □9 □10

(Does not (completely

interfere) interferes)

F. Sleep

□0 □1 □2 □3 □4 □5 □6 □7 □8 □9 □10

(Does not (completely

interfere) interferes)

G. Enjoyment of life

□0 □1 □2 □3 □4 □5 □6 □7 □8 □9 □10

(Does not (completely

interfere) interferes)

**Six-month follow-up questionnaire**

Date of (MM/DD/YY) (MM/DD/YY)

Patient Name

Patient ID

1. Throughout our lives, most of us have had pain from time to time (such as minor headaches, sprains, and toothaches). Have you had pain other than these everyday kinds of pain today?

□Yes

□No

2. On the diagram, shade in the areas where you feel pain. Put an X on the area that hurts the most.


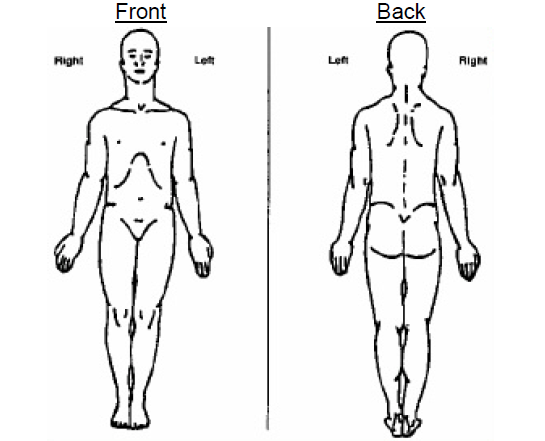


3. Please rate your pain by marking the box beside the number that best describes your pain at rest in the past week.

□0 □1 □2 □3 □4 □5 □6 □7 □8 □9 □10

(no (worst pain

pain) imaginable)

4. Please rate your pain by marking the box beside the number that best describes your pain on movement in the past week.

□0 □1 □2 □3 □4 □5 □6 □7 □8 □9 □10

(no (worst pain

pain) imaginable)

1. What treatments or medications are you receiving for your pain?

6. In the last 24 hours, how much relief have pain treatments or medications provided? Please mark the box below the percentage that most shows how much relief you have received.

□0% □10% □20% □30% □40% □50% □60% □70% □80% □90% □10%

(no (complete

relief) relief)

7. Mark the box beside the number that describes how, during the past 24 hours, pain has interfered with your:

A. General Activity

□0 □1 □2 □3 □4 □5 □6 □7 □8 □9 □10

(Does not (completely

interfere) interferes)

B. Mood

□0 □1 □2 □3 □4 □5 □6 □7 □8 □9 □10

(Does not (completely

interfere) interferes)

C. Walking ability

□0 □1 □2 □3 □4 □5 □6 □7 □8 □9 □10

(Does not (completely

interfere) interferes)

D. Normal Work (includes both work outside the home and housework)

□0 □1 □2 □3 □4 □5 □6 □7 □8 □9 □10

(Does not (completely

interfere) interferes)

E. Relations with other people

□0 □1 □2 □3 □4 □5 □6 □7 □8 □9 □10

(Does not (completely

interfere) interferes)

F. Sleep

□0 □1 □2 □3 □4 □5 □6 □7 □8 □9 □10

(Does not (completely

interfere) interferes)

G. Enjoyment of life

□0 □1 □2 □3 □4 □5 □6 □7 □8 □9 □10

(Does not (completely

interfere) interferes)

**Twelve-month follow-up questionnaire**

Date of (MM/DD/YY) (MM/DD/YY)

Patient Name

Patient ID

1. Throughout our lives, most of us have had pain from time to time (such as minor headaches, sprains, and toothaches). Have you had pain other than these everyday kinds of pain today?

□Yes

□No

2. On the diagram, shade in the areas where you feel pain. Put an X on the area that hurts the most.


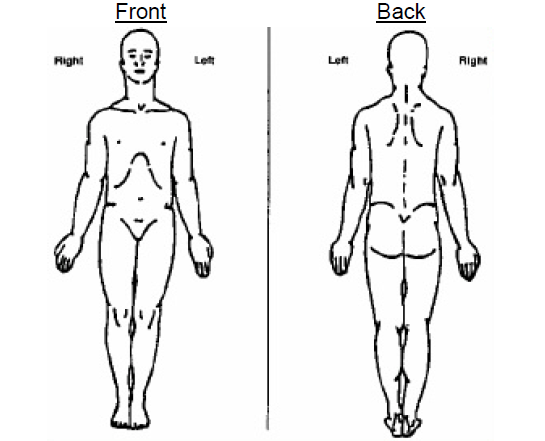


3. Please rate your pain by marking the box beside the number that best describes your pain at rest in the past week.

□0 □1 □2 □3 □4 □5 □6 □7 □8 □9 □10

(no (worst pain

pain) imaginable)

4. Please rate your pain by marking the box beside the number that best describes your pain on movement in the past week.

□0 □1 □2 □3 □4 □5 □6 □7 □8 □9 □10

(no (worst pain

pain) imaginable)

1. What treatments or medications are you receiving for your pain?

6. In the last 24 hours, how much relief have pain treatments or medications provided? Please mark the box below the percentage that most shows how much relief you have received.

□0% □10% □20% □30% □40% □50% □60% □70% □80% □90% □10%

(no (complete

relief) relief)

7. Mark the box beside the number that describes how, during the past 24 hours, pain has interfered with your:

A. General Activity

□0 □1 □2 □3 □4 □5 □6 □7 □8 □9 □10

(Does not (completely

interfere) interferes)

B. Mood

□0 □1 □2 □3 □4 □5 □6 □7 □8 □9 □10

(Does not (completely

interfere) interferes)

C. Walking ability

□0 □1 □2 □3 □4 □5 □6 □7 □8 □9 □10

(Does not (completely

interfere) interferes)

D. Normal Work (includes both work outside the home and housework)

□0 □1 □2 □3 □4 □5 □6 □7 □8 □9 □10

(Does not (completely

interfere) interferes)

E. Relations with other people

□0 □1 □2 □3 □4 □5 □6 □7 □8 □9 □10

(Does not (completely

interfere) interferes)

F. Sleep

□0 □1 □2 □3 □4 □5 □6 □7 □8 □9 □10

(Does not (completely

interfere) interferes)

G. Enjoyment of life

□0 □1 □2 □3 □4 □5 □6 □7 □8 □9 □10

(Does not (completely

interfere) interferes)
